# Treatment Outcome for Adults in a Residential Program for Binge Eating Spectrum Disorders: Protocol for a Prospective Pragmatic Single-Arm Trial

**DOI:** 10.2196/32270

**Published:** 2022-05-24

**Authors:** Renee D Rienecke, Dan V Blalock, Haley D Mills, Alan Duffy, Jamie Manwaring, Daniel Le Grange, Philip S Mehler, Susan McClanahan, Craig Johnson

**Affiliations:** 1 Eating Recovery Center Chicago, IL United States; 2 Durham Veterans Affairs Medical Center Durham, NC United States; 3 Eating Recovery Center Denver, CO United States; 4 University of California, San Francisco San Francisco, CA United States

**Keywords:** binge eating spectrum disorders, binge eating disorder, bulimia nervosa, treatment, residential program

## Abstract

**Background:**

Most studies reporting treatment outcomes for eating disorders at higher levels of care focus on anorexia nervosa and bulimia nervosa. No studies have been published with a singular focus on examining treatment outcomes for adults receiving residential programming specifically designed for the treatment of binge eating spectrum disorders (BESD), including binge eating disorder and bulimia nervosa.

**Objective:**

The purpose of this paper is to outline the protocol of a prospective study examining treatment outcomes at discharge and 3-month, 6-month, and 12-month postdischarge follow-up, for a sample of consecutive admissions to a residential program specifically for patients with BESD.

**Methods:**

One hundred consecutive admissions to a binge eating treatment program were enrolled in the prospective single-arm trial between January 2019 and February 2020. Data were collected at admission, discharge, and 3, 6, and 12 months postdischarge, with admission, discharge, and 12-month follow-up as the major timepoints of interest. Results across the major timepoints will be analyzed with mixed effects general linear models.

**Results:**

The primary aim is to assess the impact of the program on eating disordered behaviors at discharge and 12-month follow-up, which are hypothesized to improve as a result of treatment. Secondary hypotheses include improvements on comorbid symptoms, including trauma, depression, and obsessive-compulsive symptoms, as well as improvements on medical indicators of health, including cholesterol and triglycerides, at discharge and 12-month follow-up.

**Conclusions:**

This study may aid in the development of treatment guidelines for patients with BESD at higher levels of care and lend support to having specialty treatment programs for patients with BESD.

**International Registered Report Identifier (IRRID):**

DERR1-10.2196/32270

## Introduction

### Background

Binge eating disorder (BED) is defined as recurrent episodes of binge eating in the absence of compensatory behaviors, accompanied by marked distress regarding the binge eating [1]. Lifetime prevalence rates of BED among adults are 0.85%-1.9% [2,3] and are often associated with psychiatric comorbidity, medical complications, and a BMI over 30 [2,4]. Bulimia nervosa (BN) is characterized by episodes of binge eating accompanied by compensatory behaviors such as self-induced vomiting [1]. Outpatient treatment based on cognitive-behavioral therapy (CBT) is generally recommended for binge eating spectrum disorders (BESD) such as BED and BN [5,6]. There are times, however, when patients require higher levels of care, such as residential treatment, to interrupt severe and enduring eating disordered behaviors.

Residential treatment provides 24/7 care focused on psychological/behavioral interventions (as opposed to medical stabilization, which is the focus of inpatient treatment). A systematic review of 19 residential eating disorder treatment programs reported that only 14% of the reviewed studies included patients with BED [7]. In contrast, patients with anorexia nervosa (AN) were included in all 19 studies, and patients with BN were included in 90% of the studies reviewed. It is possible that results for patients with BED have not been reported as frequently as AN or BN due to BED only being a recognized diagnosis since the publication of the Diagnostic and Statistical Manual of Mental Disorders, Fifth Edition (DSM-5) in 2013 [1]. Alternatively, binge eating may be underreported due to its normalization to some degree [8].

One of the few studies reporting results separately for BED was from a residential treatment program for obesity, examining patients with and without BED at 6-month and 5-year follow-up [9]. At both follow-up points, there were no differences in weight loss between obese patients with and without BED. Patients with BED had significantly worse scores on all psychosocial measures at baseline and 6-month follow-up. Another study found significant reductions in weight after a 5-month stay at a residential eating disorder program in Italy [10].

Outcomes for patients with BN are reported more frequently than outcomes for patients with BED, although BN outcomes are often not reported separately from other eating disorder diagnoses, with results reported for the overall sample instead. Residential programs that have reported results separately for patients with BN often find stability in BMI over time and improvements in eating disorder psychopathology and depression [10-14]. Purging behaviors have been shown to decrease almost completely for patients with BN while in residential treatment [15].

Although there are no widely agreed upon essential elements of residential treatment for BED, in their review of residential programs across eating disorder diagnoses, Peckmezian and Paxton [7] found that 52% of programs used CBT as a treatment modality, whereas 33% used dialectical behavior therapy (DBT) and 19% used acceptance and commitment therapy (ACT). Briefly, CBT emphasizes behavioral change and addressing overvaluation of shape and weight [16], DBT focuses on teaching skills such as mindfulness and emotion regulation [17], and ACT encourages patients to develop psychological flexibility and live in accordance with their values and goals [18].

Most residential programs are generally designed for patients across the eating disorders spectrum. No studies have been published with a singular focus on examining treatment outcomes for adults receiving residential programming specifically designed for the treatment of BESD. The purpose of this paper is to describe the detailed protocol of a study prospectively examining treatment outcomes for a sample of consecutive admissions to a residential program specifically for patients with BESD (BED or BN). Patients with AN – binge/purge subtype (AN-BP) were not included in the binge eating disorder treatment program described in this paper, given the need for a different treatment focus for this patient population, including weight restoration.

### Primary Hypotheses

It is hypothesized that meaningful improvements (defined here as pre- to posttreatment change with at least a medium Cohen *d* effect size of 0.5) will be demonstrated on measures of eating disorder psychopathology as a result of treatment addressing binge eating and other disordered eating behaviors. It is also hypothesized that a history of trauma and current posttraumatic stress disorder (PTSD) symptoms will moderate treatment effects, in that improvements in eating disorder symptoms will be less marked among those with a history of trauma or current PTSD symptoms. Although some studies have found that PTSD does not result in poorer treatment outcomes for binge eating [19,20], other studies have found that PTSD predicts more binge eating at end of treatment [21] and premature treatment dropout for patients with BN [22]. Given these mixed findings and the assumed increased level of severity in the current population seeking higher level of care treatment, PTSD will be examined as a moderator.

### Secondary Hypotheses

It is also hypothesized that meaningful improvements will be demonstrated on a measure of experiential avoidance of weight-related feelings, thoughts, and actions, although no a priori hypotheses are made as to which subscales may improve the most. Depression, dysfunctional attitudes, and quality of life are also expected to meaningfully improve. Small improvements (defined here as pre- to posttreatment change with at least a small Cohen *d* effect size of 0.3) on a measure of obsessive-compulsive symptoms are expected, as addressing obsessive-compulsive symptoms is not a main target for treatment overall in the treatment program, but may be addressed in individual therapy. Anxiety and self-esteem are both hypothesized to meaningfully improve after treatment. Given that the treatment program incorporates trauma-informed care, it is hypothesized that meaningful improvements on a measure of PTSD symptoms will be found. It is also hypothesized that treatment will result in improved levels of triglycerides, high-density lipoprotein (HDL) and low-density lipoprotein (LDL) cholesterol, and glycated hemoglobin (HbA_1c_) due to normalization of eating patterns. It is well established that having a BMI over 30 shortens life and adversely impacts years of productive life [23]. The mechanism for those adverse effects is via contributors to the development of the metabolic syndrome and a heightened cardiac risk profile, such as hyperlipidemia and hyperglycemia. Thus, measuring those variables at baseline and then reassessing their serum values after treatment is one way to define the potential value of this program to reduce cardiac risk. Findings from this study may inform treatment guidelines for patients with BESD receiving treatment at higher levels of care.

## Methods

### Study Design

This study is a prospective pragmatic single-arm trial with consecutive admissions. As with all pragmatic trials, the primary goal of this study was to observe unbiased patient outcomes in a real-world setting. Given the acuity of patients, and the lack of an evidence-based treatment as usual for patients with BESD at this level of care, it was neither feasible nor ethical to execute a randomized or nonrandomized design with a treatment as usual or nontreatment comparison condition. To minimize bias from retrospective reports and convenience samples, this study was devised prospectively to examine outcomes in 100 consecutive admissions who agreed to participate.

### Participants and Eligibility Criteria

One hundred consecutive admissions to the Binge Eating Treatment & Recovery (BETR) residential program in a large Midwestern city in the United States who consented to participate were enrolled in this study. The BETR program is part of Eating Recovery Center’s (ERC) network of eating disorder treatment programs. ERC is a multisite, national program offering higher levels of care to patients with eating disorders. Treatment is provided by multidisciplinary teams of a physician, licensed psychotherapist, psychiatrist, and registered dietitian, with a focus on evidence-based group therapies. Participants were approached to participate in the study within the first 3 days of their admission date.

Inclusion criteria for patients admitting to the BETR treatment program are the following: (1) age ≥18 years, (2) have binge eating as a predominant symptom associated with their primary or secondary diagnosis at admission, and (3) voluntary consent for treatment from the patient. Patients were recommended for residential level of care (as opposed to partial hospitalization programming) if they had prominent mood and anxiety symptoms (ie, nonsuicidal self-injury, suicidality, sleep disturbance) associated with their eating disorder and/or if they lacked support for recovery in their home environments. Other inclusion criteria included severity of eating disorder symptoms, abnormalities in initial labs, or poorly controlled medical conditions that were exacerbated by eating disorder symptoms (ie, diabetes mellitus), sleep-related disturbances, or nocturnal-related eating. Exclusion criteria that prevented patients from admitting to the BETR treatment program are the following: (1) primary substance use or psychotic disorder, (2) active psychosis, (3) immobility (patients could not be bedbound, and needed to be able to perform activities of daily living independently or with some nursing assistance), (4) high risk for refeeding syndrome or need for refeeding secondary to restriction, due to the lack of capacity for tube feeds/lack of focus on refeeding on the unit, and (5) need for inpatient medical stabilization. Additionally, and without regard to this study, patients are always asked (but never required) to complete a battery of self-report measures at admission and discharge. An additional inclusion criterion for this study was consent to participate in this study in addition to treatment, including providing self-report data and lab draws while in treatment and during follow-up posttreatment. Exclusion criteria for the study were (1) unwillingness to provide informed consent, and (2) the presence of any intellectual disability, cognitive deficit, or physical incapacitation that may have prevented participants from understanding or completing the informed consent process or completing assessment measures.

### Study Procedures

In addition to the standard admission and discharge questionnaires, and standard treatment described below, the 100 consecutive admissions for this study were asked to follow additional data collection procedures and were reimbursed accordingly. In addition to admission and discharge questionnaires, patients were asked to complete 6-month and 12-month follow-up self-report questionnaires. Patients were also asked to visit a laboratory facility at 3-month and 6-month follow-up to obtain a blood draw for a lipid panel (total cholesterol, HDL, LDL, triglycerides) and HbA_1c_. Participants received a US $25 Amazon gift card after completion of each of their admission and discharge assessments, and a US $100 Amazon gift card after the 6-month and 12-month follow-up intervals, for a possible total of US $250 in gift cards for completing all assessments.

### Ethics Approval

This study was approved by Salus Institutional Review Board in January 2019 (approval number: ERC-001) and procedures followed were in accordance with the ethical standards of the institutional review board on human experimentation and the Helsinki Declaration of 1975.

### Treatment

The BETR program is designed to specifically serve the needs of patients struggling with BESD. A case report suggested that having patients with BESD participate in treatment programs designed primarily for restrictive eating disorders may be problematic due to differing foci of weight restoration versus normalizing eating patterns, as well as patients’ comparisons to other patients, which can be detrimental to recovery [24,25]. Although there is little empirical evidence to support this, a study of intensive treatment specifically for patients reporting episodes of overeating (patients with BN, BED, or obesity without binge eating) found improvements in eating disorder psychopathology after 5 weeks, suggesting that targeted specialty care may be more effective and more efficient than general eating disorder treatment programs that address a wide range of symptomatology [26]. In addition, patients with BESD have unique medical concerns that may best be treated in their own programming. For example, compared to healthy controls, patients with BED have been found to be 13 times more likely to develop type 2 diabetes [27]. They are also more likely to exhibit several other medical complications including insulin resistance, hypertension, acid reflux, and obstructive sleep apnea [4,28-30].

Patients receive personalized treatment from a multidisciplinary team for biweekly individual therapy, biweekly psychiatry visits, weekly family therapy, and weekly dietary sessions. Additionally, patients meet with a primary care physician, certified exercise physiologist, case manager, behavioral health counselors, and nurses upon admission and as needed. The registered dietitian and physician jointly decide on meal plans based on patients’ treatment goals and medical conditions. Patients participate in 3 supported meals and 2-3 supported snacks daily. Treatment is focused on regulating eating and treating body image disturbance. The exercise philosophy is on increasing joyful and intuitive movement, increasing mobility when needed, decreasing body shame, and decreasing overexercise or compulsive exercise when present.

In addition to interrupting eating disordered behavior, treatment also focuses on managing comorbid conditions, such as mood and anxiety disorders, and regulating sleep. Patients participate in daily group therapy based on a range of treatment modalities, including enhanced CBT (CBT-E) [16], DBT [17], ACT [18], narrative therapy, and exposure and response prevention, and on a range of topics, including body image, mood and anxiety, and nutrition. A primary focus on CBT-E and DBT was chosen because of the research evidence supporting their use for disorders characterized by binge eating. Trauma-informed care was also part of the treatment program. The average length of stay in the BETR program is 30 days, although this can vary considerably based on patients’ progress and insurance coverage.

### Measures

#### Primary Outcomes: Eating Behavior

The Eating Pathology Symptoms Inventory (EPSI) [31] is a 45-item self-report measure of eating pathology with eight subscales: body dissatisfaction, binge eating, cognitive restraint, purging, restricting, excessive exercise, negative attitudes toward obesity, and muscle building. Items are scored on a 5-point scale from 0 (never) to 4 (very often). The EPSI has been shown to have good psychometric properties [32].

The Binge Eating Scale (BES) [33] is a 16-item self-report measure assessing binge eating behaviors that may be indicative of an eating disorder. It measures eating disordered behaviors as well as feelings associated with a binge episode. Items are measured on a 4-point scale, and the measure has been shown to have good psychometric properties [34].

The Night Eating Questionnaire (NEQ) [35] is a 14-item self-report measure of various aspects of night eating, including percentage of food consumed after dinner and frequency of nocturnal awakenings and ingestion of food. Items are scored on a 5-point scale.

#### Secondary Outcomes: Comorbid Symptoms

The Acceptance and Action Questionnaire for Weight-Related Difficulties – Revised (AAQW-R) [36] is a 10-item self-report measure of experiential avoidance of weight-related feelings, thoughts, and actions with good psychometric properties. It has three subscales: food as control, weight as a barrier to living, and weight stigma [37].

The Eating Disorders Quality of Life (EDQOL) instrument [38] is a 25-item self-report measure assessing the impact of an eating disorder on one’s quality of life. It has a total score and four subscales: psychological, physical/cognitive, financial, and work/school. Items are scored on a 5-point scale from 0 (never) to 4 (always). It has been found to have good convergent and discriminant validity and good test-retest reliability [38].

The Beck Depression Inventory-II (BDI-II) [39] is a widely used 21-item self-report measure assessing the severity of depressive symptoms. Items are scored on a 4-point scale, with total scores ranging from 0-63. The measure has been found to have adequate reliability and validity [39].

The Obsessive-Compulsive Inventory – Revised (OCI-R) [40] is an 18-item self-report measure of obsessive-compulsive symptoms. It assesses six groups of symptoms, including washing, checking, ordering, obsessing, hoarding, and neutralizing. Items are scored on a 5-point scale from 0 (not at all) to 4 (extremely). It has been shown to have good internal consistency, test-retest reliability, and convergent and discriminant validity [40].

The State-Trait Anxiety Inventory (STAI) [41] is a 40-item self-report measure of state and trait anxiety, with 20 items assessing how respondents feel “at this moment,” and 20 items assessing how respondents “generally feel.” Items are scored on a 4-point scale from 1 (not at all/almost never) to 4 (very much so/almost always). Psychometric properties have been found to be adequate [41].

The Rosenberg Self-Esteem Scale (RSE) [42] is a 10-item self-report measure of self-esteem, with items scored on a 4-point scale from 1 (strongly agree) to 4 (strongly disagree). It has been found to have excellent internal consistency and test-retest reliability [42].

The Dysfunctional Attitudes Scale – Short Form (DAS-SF 1) [43] is a 9-item measure assessing dysfunctional attitudes of individuals with depression. Items are scored on a 4-point scale from “totally disagree” to “totally agree.” The measure is highly correlated with the original 40-item version of the DAS and has good internal consistency [43].

The PTSD Checklist for DSM-5 (PCL-5) [44] is a 20-item self-report measure that assesses the DSM-5 [1] symptoms of PTSD over the previous month. There are four subscales that correspond to PTSD symptom clusters B (intrusions), C (avoidance), D (negative alterations in cognitions and mood), and E (alterations in arousal and reactivity). It has demonstrated good internal consistency, test-retest reliability, and convergent and discriminant validity among veterans [45]. It has not yet been validated in an eating disorder population. The PCL-5 will be examined as an outcome, but also as a predictor variable to determine whether patients with higher PCL scores have poorer outcomes compared to those with lower PCL scores.

The Adverse Childhood Experiences Survey (ACES) [46] is a 10-item self-report measure that assesses 10 types of childhood trauma, including physical, verbal, or sexual abuse, physical or emotional neglect, having a parent who struggles with alcoholism, having a mother who is a victim of domestic violence, having a family member in jail, having a family member diagnosed with a mental illness, and having a family member unavailable due to divorce, death, or abandonment. Adverse childhood experiences (ACEs) have been found to be related to a number of psychiatric and medical illnesses in adulthood, often with a dose-response relationship wherein risk for illness in adulthood is related to a greater number of ACEs in childhood [46]. The ACES will be examined primarily as a predictor variable to determine whether patients with more ACEs have poorer outcomes compared to those with fewer or no ACEs.

### Secondary Outcomes: Medical/Physiological Variables

At 3 and 6 months postdischarge, patients obtained a blood draw for a lipid panel (total cholesterol, HDL, LDL, triglycerides) and HbA_1c_. Patients were weighed weekly or twice weekly depending on insurance requirements and symptom presentation (ie, active purging was monitored more frequently with weights and labs). All weight collection was blind. “Blind weighing” involves not sharing weight data with the patient to minimize potential distress that may occur from the patient seeing his or her weight [47]. Table 1 displays a timeline of measures and labs that were collected.

**Table 1:** Timeline of collection for labs and questionnaires.

		Baseline	Discharge	3-month follow-up	6-month follow-up	12-month follow-up
**Labs**						
	Total cholesterol	x		x	x	
	High density lipoprotein	x		x	x	
	Low density lipoprotein	x		x	x	
	Triglycerides	x		x	x	
	Glycated hemoglobin	x		x	x	
**Assessments**						
	Eating Pathology Symptoms Inventory	x	x		x	x
	State-Trait Anxiety Inventory	x	x		x	x
	Acceptance and Action Questionnaire for Weight-Related Difficulties – Revised	x	x		x	x
	Beck Depression Inventory-II	x	x		x	x
	Binge Eating Scale	x	x		x	x
	Dysfunctional Attitudes Scale – Short Form	x	x		x	x
	Eating Disorders Quality of Life	x	x		x	x
	Night Eating Questionnaire	x	x		x	x
	Obsessive-Compulsive Inventory – Revised	x	x		x	x
	PTSD Checklist for DSM-5	x	x		x	x
	Rosenberg Self-Esteem Scale	x	x		x	x

### A Priori Analytic Plan

All data at admission, discharge, and 3, 6, and 12 months postdischarge will be aggregated for the full sample and examined for patterns of missingness, outliers (>3 standard deviations from mean), and normality of distributions. Our a priori assumptions are that data will be missing at random, and all data will be normally distributed with the appropriately minimal proportion of outlier data points. Mixed models will be used to examine fixed and random effects of linear within-person change in BDI-II, EDQOL, OCI-R, STAI, RSE, DAS-SF, PCL-5, BES, and NEQ across our major timepoints of interest (admission, discharge, and 12-month follow-up). Mixed models will be run both unadjusted, as well as adjusted for person-level variables of BMI at admission, demographic variables of age, race/ethnicity, and gender, and diagnosis. Lastly, to examine the potential for different trajectories within-treatment and posttreatment, nested model comparisons will be conducted comparing the linear mixed models described above with mixed models including a quadratic time variable. Based on an expected effect size of at least *d*=0.5, and to account for a range of potentially inflated intraclass correlation coefficients that could reduce power, a sample size of 100 was determined to be sufficient to test both the mixed effects main effect hypotheses above, as well as the mixed effects 2-level interactions specified below.

All data at each of these timepoints will also be subset by patients above and below the threshold for history of trauma (defined as an ACES score ≥4) and above and below the most commonly established threshold for clinically significant levels of PTSD symptoms via the PCL-5 (PCL-5 ≥33) [48]. These subsets of patients will be compared using independent-samples *t* tests to determine any differences in point-prevalence of BDI-II, EDQOL, OCI-R, STAI, RSE, DAS-SF, BES, and NEQ by history of trauma or current trauma symptoms, and traditional moderation analyses with interaction terms added to the mixed models described above to determine any differences in changes over time of BDI-II, EDQOL, OCI-R, STAI, RSE, DAS-SF, BES, and NEQ by history of trauma or current trauma symptoms.

## Results

Recruitment of participants began in January 2019 and ended in February 2020. Data collection was completed in May 2021. Data analysis is expected to begin in April 2022, and results are expected to be published in fall 2022. The study was internally funded by ERC.

## Discussion

### Principal Findings

The treatment program described in this study is unique in that it is developed specifically for, and solely treats, patients with BESD at the residential level of care. It is expected that this program, tailored to the needs of patients with BESD, will result in improvement in eating disorder psychopathology, comorbid mood and anxiety symptoms, quality of life, and improvement in physiological measures of health as a result of changes in eating disordered behaviors.

Several practical challenges in evaluating real-world treatment make it important to develop a protocol and explicitly state a priori intentions and expectations. First, the lack of a control group makes it difficult to assess a program’s effectiveness. Nevertheless, our a priori hypotheses on the *degree* of improvement may help findings from this study aid in the development of treatment guidelines for other programs seeking to help adults with BESD, which is critically important given the increased prevalence of BESD compared to other eating disorders, and the medical complications unique to BED [30]. Second, because treatment is not conditional on consenting to provide research data, potential bias from evaluating a program based solely on voluntarily collected research data over time may occur. To mitigate this bias in this study, we prospectively created a protocol that would allow for consecutive admissions to the program to be evaluated without the need for an additional research overlay that may lead to missing data in a biased fashion. Finally, because this is the first study to examine treatment for BESD at a higher level of care, it is important to clarify ahead of time exactly what treatment is being performed, what patients are admitted to the program, and what outcomes are expected as a result of treatment in the program.

### Conclusions

This study may aid in the development of treatment guidelines for patients with BESD at higher levels of care and lend support to having specialty treatment programs for patients with BESD. Further, it may aid in improving insurance coverage for BESD, which tends to be less favorable than that for patients with AN or BN who are in treatment at ERC. Future studies should further examine components of treatment programs to determine which elements are most critical to recovery.

## Abbreviations

AAQW-R: Acceptance and Action Questionnaire for Weight-Related Difficulties – Revised
ACE: adverse childhood experience
ACES: Adverse Childhood Experiences Survey
ACT: acceptance and commitment therapy
AN: anorexia nervosa
AN-BP: anorexia nervosa – binge/purge subtype
BDI-II: Beck Depression Inventory-II
BED: binge eating disorder
BES: Binge Eating Scale
BESD: binge eating spectrum disorders
BETR: Binge Eating Treatment & Recovery
BN: bulimia nervosa
CBT: cognitive-behavioral therapy
CBT-E: enhanced cognitive-behavioral therapy
DAS-SF 1: Dysfunctional Attitudes Scale – Short Form
DBT: dialectical behavior therapy
DSM-5: Diagnostic and Statistical Manual of Mental Disorders, Fifth Edition
EDQOL: Eating Disorders Quality of Life
EPSI: Eating Pathology Symptoms Inventory
ERC: Eating Recovery Center
HbA_1c_: glycated hemoglobin
HDL: high density lipoprotein
LDL: low density lipoprotein
NEQ: Night Eating Questionnaire
OCI-R: Obsessive-Compulsive Inventory – Revised
PCL-5: PTSD Checklist for DSM-5
PTSD: posttraumatic stress disorder
RSE: Rosenberg Self-Esteem Scale
STAI: State-Trait Anxiety Inventory
